# Addressing complex societal challenges in health education – A physiotherapy-led initiative embedding inclusion health in an undergraduate curriculum

**DOI:** 10.12688/hrbopenres.12939.2

**Published:** 2020-03-06

**Authors:** Julie Broderick, Alice Waugh, Mark Mc Govern, Lucy Alpine, Sinead Kiernan, Niamh Murphy, Sofia Hodalova, Sorcha Feehan, Clíona Ní Cheallaigh

**Affiliations:** 1 Discipline of Physiotherapy, School of Medicine, Trinity College Dublin, the University of Dublin, Dublin, Ireland; 2 Department of Physiotherapy, St. James's Hospital, Dublin, Ireland; 3 School of Medicine, Trinity College Dublin, the University of Dublin, Dublin, Ireland; 4 Department of General Medicine and Infectious Diseases, St James's Hospital, Dublin, Ireland

**Keywords:** Inclusion health, homeless, homelessness, education, clinical placement, curriculum

## Abstract

People who are socially excluded experience vastly poorer health outcomes compared to the general population. Inclusion Health seeks to directly address this health inequity. Despite the increased requirement for health care and the increased prevalence of complex health and social needs in socially excluded people, Inclusion Health features very little in health education curricula.

This letter has been written by a group of clinicians, academics, clinical education specialists and students with a common interest in Inclusion Health. In the absence of established guidance on how best to incorporate the broad topic of inclusion health in undergraduate education, we have developed a two-pronged approach within Physiotherapy. We are writing to highlight the following initiatives; firstly, the provision of a dedicated undergraduate clinical placement devoted to the area of Inclusion Health. Secondly, we have also initiated a step-wise process of introducing the topic of Inclusion Health into the formal undergraduate curriculum.

This letter demonstrates the need to implement strategies to incorporate Inclusion Health into the curriculum and the approaches described are applicable to diverse health professions and settings.

## Disclaimer

The views expressed in this article are those of the authors. Publication in HRB Open Research does not imply endorsement by the Health Research Board of Ireland.

## Introduction

Inclusion Health is an approach that aims to address the extreme health inequalities experienced by socially excluded people ^1^. Social exclusion is linked with poverty and low income, but is further characterised by poor access to employment, education, housing, health services and experiences of crime, incarceration and family breakdown. These experiences are linked, and often form part of an intergenerational cycle ^2, 3^. People with substance use disorders, prisoners, casual sex-workers, as well as Travellers and Aboriginal/First Nations people, frequently experience social exclusion ^4, 5^. The prevalence of multiple traumas or adverse events in childhood and adulthood is much higher in socially excluded people than the general population ^6^. Social exclusion is also associated with increased morbidity and mortality similar to seen with that associated with poverty, but of a much higher magnitude ^3^. Socially excluded people have a standardised mortality rate eight times higher than the average for men, and nearly twelve times higher for women ^4^.

Homelessness predominantly affects people who have already experienced social exclusion and adversity since childhood, and compounds the effect of social exclusion on health ^6– 8^. In Ireland, a shortage of public housing has led to homelessness becoming a national crisis ^9^. Homeless people are predominantly located in the capital city, Dublin. Since 2013, the number of adults who are homeless in Dublin has doubled ^9^. Homeless people in Ireland have rates of poor physical and mental health, chronic disease, and multimorbidity which are greatly increased compared to the general population ^10^. A similar phenomenon has been reported in other high-income countries ^11^. The median age at death for people experiencing homelessness in Dublin is devastatingly low at 44 years for males and 36 years for females ^12^.

Unsurprisingly, given high rates of physical and mental ill-health, homelessness is associated with increased usage of unscheduled health care. The centrally located Dublin hospitals, including St. James’s Hospital, have seen increasing prevalence of homelessness in patients. The effect of homelessness on usage of acute unscheduled hospital care is demonstrated in the recent report that, despite representing only 0.4% of the catchment people experiencing homelessness account for almost 10% of emergency department attendances and inpatient stays in St James’s Hospital ^13^. Homeless people, therefore, represent a significant proportion of patients requiring hospital care in St James’s Hospital.

People experiencing homelessness and/or other forms of social exclusion may present challenges to clinical care providers ^14^. In light of this, there is a growing appreciation for the need for cultural and structural competence to provide care to people experiencing homelessness and/or other forms of social exclusion. Cultural competence enables healthcare providers to provide care which accommodates differences in language and/or culture ^15, 16^. Structural competence has been defined as the trained ability to discern and address clinical presentations and individual behaviors which represent downstream implications of upstream socioeconomic, political, and institutional realities ^17^. Structural competence promotes identifying and addressing stigma and aims to lead to advocacy as a means of adressing structural determinants of health. An example of cultural competency is clinician awareness of low levels of functional literacy in people experiencing homelessness resulting in an ability to provide verbal information suitable for someone with a low level of literacy. An example of structural competence would be awareness of the role of internalized and externalized stigma experienced by homeless people resulting in development of an outreach physiotherapy programme to be delivered in a setting which is more acceptable to homeless adults. Contact on an individual level between providers and patients belonging to different societal groups has been proposed to improve cultural and structural competency and to reduce stigma and bias ^18^.

Undergraduate programmes for health care providers offer an opportunity to improve provider competency in providing care to socially excluded people ^19– 21^. However, integration of Inclusion Health in medical and allied health undergraduate curricula is often lacking and/or unmeasured ^21, 22^. We suggest a formal approach should be taken. This letter describes the development of an Inclusion Health placement and a step-wise method of introducing this topic into the undergraduate Physiotherapy curriculum in Trinity College, Dublin.

## Inclusion health clinical placement

St James’s Hospital has developed an integrated, interdisciplinary Inclusion Health team, with an initial focus on homeless adults ^23^. The Inclusion Health team had noted that many of the homeless inpatients required physiotherapy. This opinion was supported by a report on the high incidence of frailty in long-term homeless adults in the catchment area ^24^. Informal discussions between the Inclusion Health team and clinical physiotherapists in the hospital, facilitated by the coach on the Quality Improvement project supporting the Inclusion Health team (a senior physiotherapist in the hospital), led to an awareness in the clinical and academic Physiotherapy Departments of the need for expertise on Inclusion Health in physiotherapists. This awareness prompted the development of a pilot Inclusion Health placement for undergraduate physiotherapy students.

A dedicated four-week pilot Inclusion Health placement was designed by key stakeholders in the Physiotherapy Department in Trinity College, Dublin (TCD) and the Physiotherapy Department and Inclusion Health team in St James’s Hospital (SJH). Key aims of the placement were to provide contact with homeless patients and to observe the provision of Inclusion Health care in the hospital and in the community. The placement included three key elements. The first element was observation of inpatient consult rounds, outpatient clinics, and weekly interagency case management meetings with the SJH Inclusion Health team, and observation of a general practitioner (GP) clinic for refugees and a GP clinic for Roma people. The second element was supervised clinical practice with homeless inpatients, and included the provision of clinical physiotherapy assessments and treatments under the supervision of clinical tutors for an average of 3.5 days per week over the 4 week duration of the placement. The third element of the placement was supervised practice with a group of homeless adults in the community, and consisted of the design, set-up, and delivery of a weekly hour-long exercise class for approximately ten homeless adults in in a residential hostel in the local area

Two third-year undergraduate physiotherapy students of Trinity College Dublin completed the placement in June-July 2019 (SF and SH). The students were supervised by two clinical tutors who were senior clinicians with a dedicated role in clinical education (MMcG and AW). Informal written/verbal feedback about the placement was sought from the two students, the two clinical tutors, the inclusion health MDT and at some of the outreach clinics attended by the students.The students completed written post-placement reflections and compiled a guidance document for future students embarking on an Inclusion Health placement. Feedback was not anonymised.

Students and facilitators reported a number of considerations to inform setting up a clinical experience within the Inclusion Health area, which are applicable to future placements/clinical exposures. These are shown in Table 1. The guidance provided by students SH and SF for future students is presented in Table 2.

**Table 1.** Key success factors for an inclusion health placement.

Key consideration	Reason
Conduct comprehensive orientation	This will maximise student confidence and preparedness, to optimise the learning experience
Allow additional time for placement planning	A comprehensive placement will consist of clinical exposure to off-site clinics and services to ensure breadth of inclusion health area is covered. Take time to build relationships with other staff and services in the area so students can be facilitated on placement
Consider interpersonal skills of students	Strong communication skills, a flexible open approach, and the ability to cope with the potentially unpredictable nature of the placement at times are needed
Peer placement recommended	A peer placement is recommended with two students placed together enabling students to undertake assessments and treatments together. Having two students together is also useful due to a number of reasons including high prevalence of physical disabilities necessitating >1 therapists to safely conduct assessments and treatments, and ability to debrief after encounters
Modify assessment	It is recommended that placement be graded on a pass/fail basis rather than a numerical grade as not all areas may be applicable to the standardised method of clinical assessment
Ensure sufficient support available for students	Due to the complexity of the area ensure there is sufficient support from senior clinicians/dedicated clinical education specialists
Arrange additional training	As the incidence of infectious diseases may be higher in inclusion health patients (for example higher incidence of HIV and Hepatitis C), infection control measures will need to be reiterated prior to commencing placement. Although the actual risk of injury is low, violence and aggression training prior to placement is recommended, mainly to learn de-escalation skills

**Table 2.** Advice from students, ‘the student voice’.

Key advice	Reason
Be empathetic & have an open mind	Have an awareness of the complex nature and difficult background of some of these patients and their co-morbidities. Speak to the nursing staff to ascertain if it is the appropriate time for treatment as patient’s mood and health status may fluctuate and will give you an indication of whether a patient is likely to engage with treatment at that time.
Communication skills are very important	Take time to build a rapport with your patient first. Assessment or treatment may need to be very brief, just talking to a patient & giving them advice may be the only treatment you will provide.
Know when not to intervene (patient unwell or not willing to engage, patient not listening to your advice)	If a patient is not on the ward or unavailable, try to get back later that day. If a patient becomes agitated or refuses to listen to your advice, it is best that you step back and try revisit the topic another day.
Plan in advance use a flexible approach	Have a plan before seeing a patient as to what you would like to get out of the session, but bear in mind that what you plan might not be what the patient would like to do. Ask the patient what they would like to get out the session/ what their priority is.
Speak to your senior clinician/practice tutor if you feel uncomfortable or unsure about a situation	Know that you should never feel out of your comfort zone when completing this placement. Speak to your senior clinician or practice tutor. Your peer is also someone you can speak to & reflect with about patients you have seen together.
You are in a safe learning environment. Generally, this patient cohort is approachable & happy to interact with students	From our experience, patients generally engaged well with assessments and treatments delivered by physiotherapy students & felt they were listened to. This patient cohort has multiple physical needs & were very receptive to input from therapies to help them.

A total of 13 inpatients and 10 outpatients were assessed and treated by students during the placement, some of whom were treated multiple times over the 4 week placement. Nine people attended the exercise classes in the residential facility with an average weekly attendance of 4 residents. Data was not recorded on the number of patients who refused or participated in assessments/treatments.

The students and clinicians involved in the placement reported bi-directional positivity – of the students towards this placement and client group and of homeless clients towards meaningful engagement and cooperation with student physiotherapists. All clinicians involved in the placement reported that it was feasible and acceptable to themselves and, as observed, to socially excluded patients. Both students reported the placement was a valuable learning opportunity. We are planning to run this placement again in the next academic year 2019–2020. We plan to ensure adequate exposure to socially excluded groups which may have different cultural needs by specifically including contact with refugees and Travellers in the placement. Space constraints for placement with the hospital Inclusion Health team and in Inclusion Health settings in the community mean that this may not be feasible for all students in our institution.

## Curricular changes

**Figure 1.**
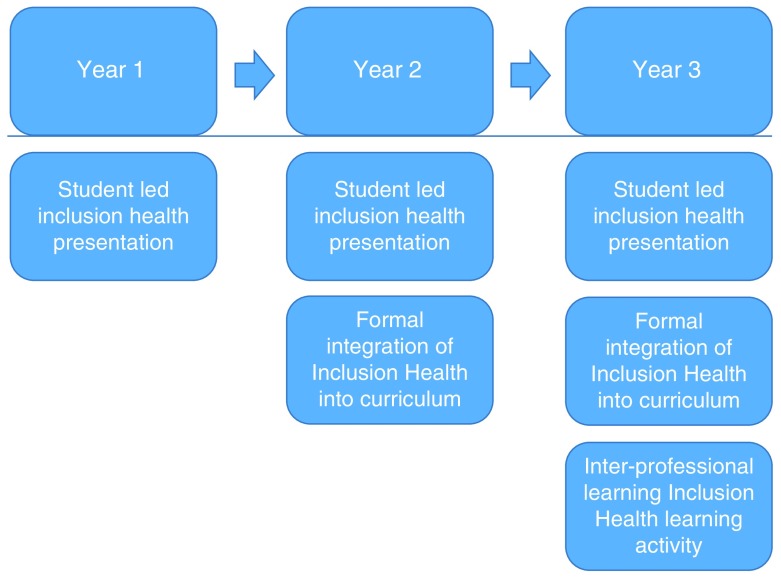
Step-wise approach of developing suite of inclusion health learning activities from Year 1 (Academic year 2018–2019) projected to Year 2 (Academic Year 2019–2020) and Year 3 (2020–2021) into formal undergraduate curriculum.

Inclusion Health is a complex topic which overlaps with mental and physical disease, as well as substance abuse and structural factors underlying the effect of social exclusion on health. An additional challenge to introducing it into the formal undergraduate curriculum is the lack of best practice guidelines or set of competencies. As demonstrated in Figure. 1, our approach is to introduce this topic in a step-wise fashion.

In the academic year 2018–2019, we commenced with group-based student-led presentations on Inclusion health. Groups were assigned titles including ‘’Inclusion Health and physiotherapy in the homeless community’’ and ‘’The role of physiotherapy in people who are asylum seekers/refugees, including special considerations post torture’’. In addition to the title, students were signposted to key resource materials and subtopics to consider for inclusion in their presentations.

In 2019–2020, the topic of Inclusion Health will be formally integrated into a pre-existing learning module (a specialist rehabilitation module, delivered in third year) and to the curriculum document. Evaluation of Inclusion Health learning outcomes will take place in formal summative assessments. In addition to the student-led presentation, there will be scheduled lectures on Inclusion Health, including homeless, traveller and refugee/asylum seeker health. In 2020–2021 an inter-professional learning activity, which is under development, will include third year students of 2–3 different health science professional students and will complement the suite of learning activities.

## Conclusion

Clinical practicum and curricula should be realigned to meet the needs of the 21 ^st^ century of which the health of socially excluded groups is a pressing need. This letter describes a unique initiative to incorporating the topic of Inclusion Health in an undergraduate physiotherapy programme via development of a four-week elective clinical placement and integration into the formal undergraduate curriculum. Our experience, albeit with a small sample (two students) and with non-anonymised feedback, suggests that this is feasible and acceptable to students, service providers and patients.

Delivery of a universal basic level of knowledge and formal integration of Inclusion Health into the undergraduate curriculum as described in this letter would ensure all students are exposed to this topic with the aim to equip all future graduates with the skills and knowledge base to work with vulnerable and complex socially excluded people to optimise health outcomes.

For deeper understanding, a dedicated clinical practicum or clinical placement including contact with a number of socially excluded people in a variety of settings is ideal. A dedicated Inclusion Health placement is likely to engage students as communication catalysts and agents of change in the health care delivery system of the future. These students may share their experiential learning with other students, professionals, educators and health care institutes; thereby enhancing future engagement from a wide range of professionals with socially excluded people.

We suggest the approach of a dedicated clinical exposure and formal integration into the curriculum could be rolled out to other health care students and applied pragmatically to other settings based on local needs and expertise.

## Data availability

### Underlying data

No data are associated with this article.
