# Supplementary material for: Addressing complex societal challenges in health education – A physiotherapy-led initiative embedding inclusion health in an undergraduate curriculum
Source: HRB Open Res. 2020 Mar 6;2:22. Originally published 2019 Sep 2. [Version 2] doi: 10.12688/hrbopenres.12939.2 (PMC6973525; doi:10.12688/hrbopenres.12939.2)
Supplement: Peer review history

# Peer review: Reviewer response for version 1

**DOI:** 10.21956/hrbopenres.14019.r26787

**Published:** 2019-09-30

**Authors:** Joseph G. McVeigh

**Affiliations:** 1 Head of Discipline of Physiotherapy, School of Clinical Therapies, College of Medicine and Health, University College Cork, Cork, Ireland

Thank you for the opportunity to review this open letter 'Addressing complex societal challenges in health education – A physiotherapy-led initiative embedding inclusion health in an undergraduate curriculum'. In this letter the authors argue for the need to implement strategies to address the needs of marginalized groups in the undergraduate physiotherapy curriculum. Specifically the letter describes the development of a physiotherapy clinical placement focused on the needs of socially marginalized groups such as homeless people, refugees etc. The letter also outlines some developments of the physiotherapy curriculum to include student led presentations and scheduled lectures on inclusion health. Future multidisciplinary/inter-professional learning developments are also mentioned.

 Marginalized groups are poorly served by health care providers, the initiative described in this letter captures an important curricular development for physiotherapy and other Allied Health Professionals. 

 It would be useful to link the developments to the CORU Standards of Proficiency for Physiotherapists.

 It would be helpful if the authors could give some indication of the number of students per year who experience the described placement.

 Some further description of what future plans are e.g. how will the inter-professional learning activity mentioned be structured.

I confirm that I have read this submission and believe that I have an appropriate level of expertise to confirm that it is of an acceptable scientific standard.

# Peer review: Reviewer response for version 1

**DOI:** 10.21956/hrbopenres.14019.r26786

**Published:** 2019-09-30

**Authors:** Luke Johnson, Serena Luchenski

**Affiliations:** 1 Institute of Epidemiology and Health care, University College London, London, UK

Dear Professor Broderick and Colleagues,

 This peer review is in response to your recent open letter, “Addressing complex societal challenges in health education – A physiotherapy-led initiative embedding inclusion health in an undergraduate curriculum.” Your letter articulates the importance of inclusion health, an emerging discipline that seeks to prevent and redress the most extreme forms of health inequities among some of the most vulnerable and underserved members of society. It goes on to advocate for and describe novel clinical educational initiatives to train future clinicians working with inclusion health groups in the field of physiotherapy. It is highly impressive and inspiring that you have taken this on at your University – thank you for sharing your experience with us to encourage others to evolve their curricula in a similar manner.

 There are two main areas for the authors to address to strengthen this article. Firstly, the section on feedback obtained for future considerations and advice on running the programme needs further expansion and detail. Additional description of the data that these are derived from, how you used that data to develop the considerations and advice that you present, and the limitations of this approach would greatly bolster the arguments you present. Secondly, the clarity of the writing and structure of the article needs improvement. We have detailed areas to consider changing below.

**1. The following section needs additional detail on the methodology, interpretation and limitations:**

Inclusion Health clinical placement

***“We found there were additional considerations to setting up a clinical experience within the inclusion health area, which are applicable to future placements/clinical exposures. These are shown in Table 1. The ‘student voice’ - physiotherapy student post-placement reflections and guidance specifically for future students embarking on an inclusion health placement are presented in Table 2.”***

 This section needs the most work. The feedback or ‘results’ you collected after running the event need to show their validity for us to take note of them. We need far more detail on how you came up with these considerations in Table 1 and advice in Table 2. For example, how were there additional considerations decided? Who came up with them? What data did they use to come up with them? How was student feedback ascertained? Was it a questionnaire? If so, how was the questionnaire designed? Was it pre-tested? Or were these reflective assignments the students had to do? Were they given guidance if so? Were these marked pieces? What was the response rate for feedback from students? Was it anonymous to minimise bias? What else was done to minimise bias? How many/what proportion of students suggested each piece of advice? How was this advice extracted from the feedback provided by the students? How did you ensure quality and validity in extracting the considerations and advice – e.g. data credibility, transferability, dependability and confirmability of findings? A section is needed towards the end of the article explicitly discussing the limitations from the feedback you have obtained too.

   

**2. The following specific areas need addressing to improve the clarity of the letter:**

Introduction

***“Socially excluded populations have a mortality rate eight times higher than the average for men, and nearly 12 times higher for women (Aldridge et al., 2018).”***

 Write ’12’ as ‘twelve’ instead to keep consistency within the sentence.

***“In Ireland, a shortage of public housing has led to homelessness becoming a national crisis.”***

 This statement needs citing.

***“Homelessness has predominantly affected people who have already experienced social exclusion and adversity since childhood, and this compounds the effect of social exclusion on health.”***

 Both statements in this sentence need citing, either as one citation which covers both statements, or two separate citations.

***“Since 2013, the number of homeless adults in Dublin has doubled (Focus Ireland).”***

 Reference ‘Focus Ireland’ needs a date too. Also, the link for this reference should be to the specific page for the source material, not just to the website front page.

***“In other areas country wide with high levels of deprivation, health care professionals will see homeless patients presenting across multiple services.”***

 Please rephrase this sentence for clarity.

Inclusion Health clinical placement

***“The curricula of higher education instituted need to reflect these changing requirements for healthcare delivery (Dean et al., 2009; McMahon et al., 2016).”***

 Statement might be stronger in the active rather than passive voice, and the word usage of “instituted” is confusing in this context – you might want to consider omitting it or changing it to “implemented”

***“Exposure of undergraduate students to patients from socially excluded groups is necessary so that graduates can become empathetic advocates and effective and innovative clinicians to help drive better health outcomes for vulnerable groups.”***

 Change the ‘and’ to ‘as well as’ to clearly divide the two characteristics.

***“We suggest a formal approach should be taken and this letter will describe the development of an inclusion health placement and a step wise method of introducing this topic into the undergraduate curriculum which we have initiated in our setting.”***

 ‘Step wise’ should be ‘step-wise’ for consistency throughout article.

***“The team had reported a high incidence of frailty in homeless adults in the catchment area (de Paul, 2017), and had noted a high rate of need for physiotherapy in homeless inpatients referred to the service”***

 Should be ‘The team has reported’ not ‘The team had reported’. Consider changing ‘and had noted a high rate of need for physiotherapy’ to ‘and noted a high need for physiotherapy’. Also, the citation ‘de Paul, 2017’ citation either needs to be moved to the end of this sentence, or a second citation needs to be added after the next claim.

***“Ongoing collaboration with the clinical and academic physiotherapy department led to the development of an inclusion health placement for undergraduate physiotherapy students.”***

 State this as more of a responsive action to the previous statement so that they are linked.

***“We report the design and roll out of a dedicated four-week inclusion health placement delivered to 3rd year undergraduate physiotherapy students of Trinity College Dublin in Jun-July 2019 on a pilot basis.”***

 You don’t need to reintroduce what you’re already reporting with ‘we report the design and roll out of’ – it’s already clear by this stage. Change ‘3 ^rd^ year’ to ‘third year’. Please state how many students were involved in the pilot.

***“Students were supervised by two clinical tutors who were senior clinicians with a dedicated role in clinical education. The main client group were homeless in-patients of St. James’s Hospital who were referred due to diverse physical and mobility limitations. Students assessed and treated this group, facilitated by the clinical tutor. Another facet of the placement was the design, set-up and delivery, of a student led exercise class in a residential hostel for homeless adults in the local area. Students also attended GP-led clinics for refugees in direct provision and a dedicated clinic for Roma people in an observational capacity.”***

 This could be restructured to be clearer/easier to read - perhaps in a list format of the different activities which students were involved with. E.g. the clinical placement involved three different facets. Firstly… Secondly… Finally.

***“The main client group were homeless in-patients of St. James’s Hospital who were referred due to diverse physical and mobility limitations”***

 You’ve used ‘inpatients’ before not ‘in-patients’; try to keep consistency and use either “inpatients” or “in-patients” but not both.

***“Students assessed and treated this group, facilitated by the clinical tutor”***

 How long did each student spend in this activity?

***“Another facet of the placement was the design, set-up and delivery, of a student led exercise class in a residential hostel for homeless adults in the local area.”***

 What assistance were students given in organising this? How many homeless people attended the event? Also, should be ‘student-led’ instead of ‘student led’.

***“Students also attended GP-led clinics for refugees in direct provision and a dedicated clinic for Roma people in an observational capacity.”***

 How many GP-led clinics did each student attend? And how many clinics for Roma people? Also, what do you mean by ‘direct provision’? I think you mean they provided physiotherapy at the GP-led clinic for refugees. This could be rephrased to be clearer.

***“Strong communication skills, a flexible open approach, and the ability to cope with the potentially unpredictable nature of the placement at times are needed”***

 How are you going to ensure students have this?

***“Due to the complexity of the area ensure there is sufficient support from senior clinicians/dedicated clinical education specialists”***

 How are you going to ensure there is enough support from staff?

***“Plan in advance use a flexible approach”***

 Do you need an ‘and’ in between these two pieces of advice?

***“This inclusion health placement demonstrated bi-directional positivity – of the students towards this placement and client group and of homeless clients towards meaningful engagement and cooperation with student physiotherapists”***

 Can you evidence this for us? Do you have feedback from students and homeless people (either quantitative or qualitative) to suggest this?

***“Students reported this was a valuable learning opportunity”***

 Again, please show feedback to evidence this.

***“We are planning to run this placement again in the next academic year 2019–2010 with an increased focus on other socially excluded groups such as refugees and travellers.”***

 Any idea how you would like to carry this out? What do you hope including these additional inclusions health populations would add in value to the students’ experience?

Curricular changes

***Figure 1***

 The boxes in your diagram should have a hyphen to say ‘student-led’ instead of ‘student led’. Also, is the box at the top of the third column meant to say ‘year 3’ instead of ‘year 2’?

***“In the academic year 2018–2019, we commenced with group-based student led presentations on the topic of inclusion health, entitled ‘’Inclusion health and physiotherapy in the homeless community’’***

 You should have a hyphen to say ‘student-led’ instead of ‘student led’?

***‘’Role of physiotherapy in people who are asylum seekers/refugees, including special considerations post torture’’***

 You should have a hyphen to say ‘post-torture’ instead of ‘post torture’.

***“Students were given the topic as well and signposted to key resource materials and subtopics to consider for inclusion in their presentations”***

 Please rephrase for clarity.

***“In 2019–2020, the topic of inclusion health will be formally integrated into a pre-existing learning module (a specialist rehabilitation module, delivered in 3rd year) and to the curriculum document.”***

 Should be ‘third year’ not ‘3rd year’.

***“In 2020–2021 an inter-professional learning activity, which is under development, will include 2–3 diverse allied health professional students and will complement the suite of learning activities.”***

 Rephrase this for clarity. Also, it would be interesting to know if you have an idea of what this activity would involve, and which allied health professional groups you would want to collaborate with?

Conclusion

***“This letter describes a unique initiative to incorporating the topic of inclusion health in an undergraduate physiotherapy programme via development of a 4-week elective clinical placement and integration into the formal undergraduate curriculum.”***

 Should be ‘four-week’ instead of ‘4-week’.

***“Delivery of a universal basic level of knowledge and formal integration of inclusion health into the undergraduate curriculum as described in this letter would ensure all students are exposed to this topic with the aim to equip all future graduates with the skills and knowledge base to work with this vulnerable and complex group to optimise health outcomes.”***

 Needs rephrasing for clarity. Consider splitting this up into two separate sentences.

***“For deeper understanding a dedicated clinical practicum or clinical placement would be recommended.”***

 Should be “For a deeper understanding”. Also, consider rephrasing the sentence from passive to active voice for a stronger concluding thought i.e. “we would recommend”.

***“Due to the nature of the area it would not be feasible to offer this opportunity to all students.”***

  What do you mean by this? Why wouldn’t it be feasible? This is a new statement and so best not introduced in the conclusion, which should just be a summary of everything discussed in the main body of the text with thoughts on what should happen next. The conclusion is not the right place to address feasibility concerns. I would either more directly address these before the conclusion in their own paragraph - with recommendations on how to address them - or eliminate this statement altogether. It takes away from the strength of your conclusion.

***“Notwithstanding this, a dedicated inclusion health placement is likely to engage students as agents of change in the health care delivery system who can be communication catalysts and agents of change for the future.”***

 You have ‘agents of change’ twice in one sentence – remove one of these.

 We hope these suggestions are useful and look forward to reading up the updated article.

 Dr Luke Johnson and Ms Serena Luchenski, FFPH

We confirm that we have read this submission and believe that we have an appropriate level of expertise to confirm that it is of an acceptable scientific standard, however we have significant reservations, as outlined above.

# Peer review: Reviewer response for version 1

**DOI:** 10.21956/hrbopenres.14019.r26789

**Published:** 2019-09-16

**Authors:** Patrick O' Donnell

**Affiliations:** 1 Graduate Entry Medical School, University of Limerick, Limerick, Ireland

This is a well written and timely description of a novel initiative developed to introduce trainee healthcare professionals to concepts and situations that are sometimes considered challenging. It highlights well the Irish context and the gravity of problems we have where social exclusion and health care concerned.  

 Suggestions:

- Consider person-centred language throughout e.g. people who are homeless, people who are socially excluded http://www.homelesshouston.org/homelessness-101-person-centered-language/ 
- The line "Homeless and other socially excluded people represent a distinct and significant population" could be altered - this gives the impression they are quite a homogenous group when we know this is not the case.
- Consider referencing Allport's contact hypothesis when describing purposes of the placement https://www.sciencedirect.com/topics/psychology/contact-hypothesis.
- For Table 1 the 'key considerations' - it is unclear to me if these are your findings or if they have been gleaned from existing literature.
- Table 1 - the term 'peer placement' is confusing.
- Table 1 - in the 'reason' column, can these be referenced to add weight?
- Table 2 - very nice to include this.

I confirm that I have read this submission and believe that I have an appropriate level of expertise to confirm that it is of an acceptable scientific standard.

# Response

**Published:** 2019-09-25

**Authors:** Julie Broderick

**Affiliations:** Trinity College Dublin, Ireland

Dear Dr. O' Donnell, 

We would like to thank you for your very insightful and valuable comments. We will certainly take those comments on board and very much appreciate your time in reviewing this article. 

Kind Regards, 

Julie Broderick and co-authors
